# Engineering Frustrated Lewis Pair Active Sites in Porous Organic Scaffolds for Catalytic CO_2_ Hydrogenation

**DOI:** 10.1021/jacs.4c01890

**Published:** 2024-05-30

**Authors:** Shubhajit Das, Ruben Laplaza, J. Terence Blaskovits, Clémence Corminboeuf

**Affiliations:** † Laboratory for Computational Molecular Design, Institute of Chemical Sciences and Engineering, École Polytechnique Fédérale de Lausanne (EPFL), 1015 Lausanne, Switzerland; ‡ National Center for Competence in Research-Catalysis (NCCR-Catalysis), École Polytechnique Fédérale de Lausanne, 1015 Lausanne, Switzerland

## Abstract

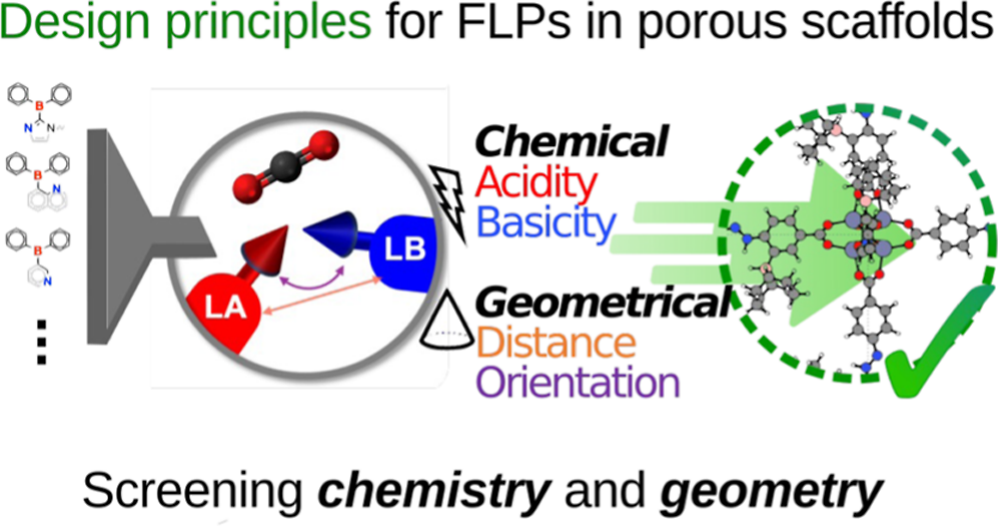

Frustrated Lewis pairs (FLPs), featuring reactive combinations of Lewis acids and Lewis bases, have been utilized for myriad metal-free homogeneous catalytic processes. Immobilizing the active Lewis sites to a solid support, especially to porous scaffolds, has shown great potential to ameliorate FLP catalysis by circumventing some of its inherent drawbacks, such as poor product separation and catalyst recyclability. Nevertheless, designing immobilized Lewis pair active sites (LPASs) is challenging due to the requirement of placing the donor and acceptor centers in appropriate geometric arrangements while maintaining the necessary chemical environment to perform catalysis, and clear design rules have not yet been established. In this work, we formulate simple guidelines to build highly active LPASs for direct catalytic hydrogenation of CO_2_ through a large-scale screening of a diverse library of 25,000 immobilized FLPs. The library is built by introducing boron-containing acidic sites in the vicinity of the existing basic nitrogen sites of the organic linkers of metal–organic frameworks collected in a “top-down” fashion from the CoRE MOF 2019 database. The chemical and geometrical appropriateness of these LPASs for CO_2_ hydrogenation is determined by evaluating a series of simple descriptors representing the intrinsic strength (acidity and basicity) of the components and their spatial arrangement in the active sites. Analysis of the leading candidates enables the formulation of pragmatic and experimentally relevant design principles which constitute the starting point for further exploration of FLP-based catalysts for the reduction of CO_2_.

## 1 Introduction

The creation of tailored catalytic active sites to drive a specific chemical transformation is an attractive strategy to design powerful catalysts for sustainable chemical production. The acid–base-nucleophile triad in hydrolase and transferase enzymes,^1^ the solid acid pores for hydrocarbon cracking in zeolites,^2^ and the nanostructures of electro- and photocatalysts^3^ all exemplify local multifunctional environments precisely tailored to minimize the energy requirement of otherwise unlikely transformations. Frustrated Lewis pairs (FLPs), a recent addition to the library of synthetic catalysts, exploit bifunctional environments to drive many catalytic transformations.^4−9^ Their unique reactivity is derived from the physical proximity of the Lewis acid (LA) and base (LB) components, in which dative quenching is prevented through constraints of a steric (substituent-based) or geometric (backbone design) nature. Since their inception as molecular catalysts in 2007, the past decade has witnessed tremendous growth in this research area, and the FLP concept has been applied to a broad class of materials including metal/covalent organic frameworks,^10−17^ zeolites,^18^ mesoporous silica,^19^ and metal oxide surfaces.^20−22^

During the last five years, the incorporation of acid–base components to the rigid backbones of heterogeneous porous scaffolds has emerged as a promising strategy to overcome the inherent drawbacks of molecular FLP catalysts, in terms of stability and recyclability. Several recent reports demonstrated in situ catalytically active semi-immobilized FLP sites, featuring the combination of one fixed Lewis component embedded in the framework of a host material and another complementary mobile component.^10−14,23−25^ In most of these studies, the organic building blocks of porous materials, primarily composed of lighter main group elements (H, B, C, N, O, etc.), are a synthetically relevant starting point for the engineering of such sites. This is due to their structural constituents being similar to those of the most frequently used LAs and LBs in molecular FLP chemistry. Nevertheless, semi-immobilized systems, in principle, could still suffer from recyclability issues owing to one of the catalytic components being soluble. Full immobilization of both the acid and base centers is arguably the most attractive conceptual approach to mitigate this problem. However, this is no trivial task due to the requirement of placing both the acid and base centers in an appropriate geometric position while maintaining the necessary chemical environment of the Lewis pairs to catalyze a given reaction of interest.^26,27^ The identification of the most promising active site architectures with appropriate chemical and geometrical compositions would thus be invaluable as a starting point for constructing such hybrid active sites. Although the chemical and geometric compositions of the FLPs have been individually linked to their performance,^26,27^ a clear connection between the full composition of active sites and the resulting catalytic performance is currently lacking. Analysis of a broader set of highly active FLP environments will guide the design of catalytic sites and realize the potential of a fully immobilized catalyst design approach.

Among the various types of porous materials, metal–organic frameworks (MOFs) offer a versatile platform as host materials for FLP sites by virtue of their well-established chemistry and synthetic tunability.^10−17^ Here, we exploit an experimental database of MOFs to engineer highly active FLP environments for direct catalytic CO_2_ hydrogenation to formate (CHTF), an important reaction from both an energy and environmental perspective.^28−31^ Note that, although stoichiometric FLP-mediated CHTF was first experimentally realized by Ashley et al. in 2009,^32^ catalytic turnovers were only reported very recently.^26,33^ Nitrogen-containing units, particularly pyridine, pyrazoles, imidazoles, triazoles, tetrazoles, and azo moieties, are widespread motifs in the organic linkers of MOFs and they constitute an existing source of chemically and geometrically diverse Lewis-basic environments to engage in FLP chemistry. These basic nitrogen sites on the organic building blocks of MOFs are utilized to generate all possible FLP environments around them by introducing acidic boron sites in a systematic manner, leading to thousands of potential B/N Lewis pair active sites (LPASs). The chemical and geometrical appropriateness of the generated LPASs are determined and the most promising active site compositions are identified. Analysis of the prospective candidates provides guiding principles for the design of optimal FLP active sites for the reduction of CO_2_.

## 2 Results and Discussion

### 2.1 Library of LPASs

#### 2.1.1 LPAS Curation

**Figure 1:**
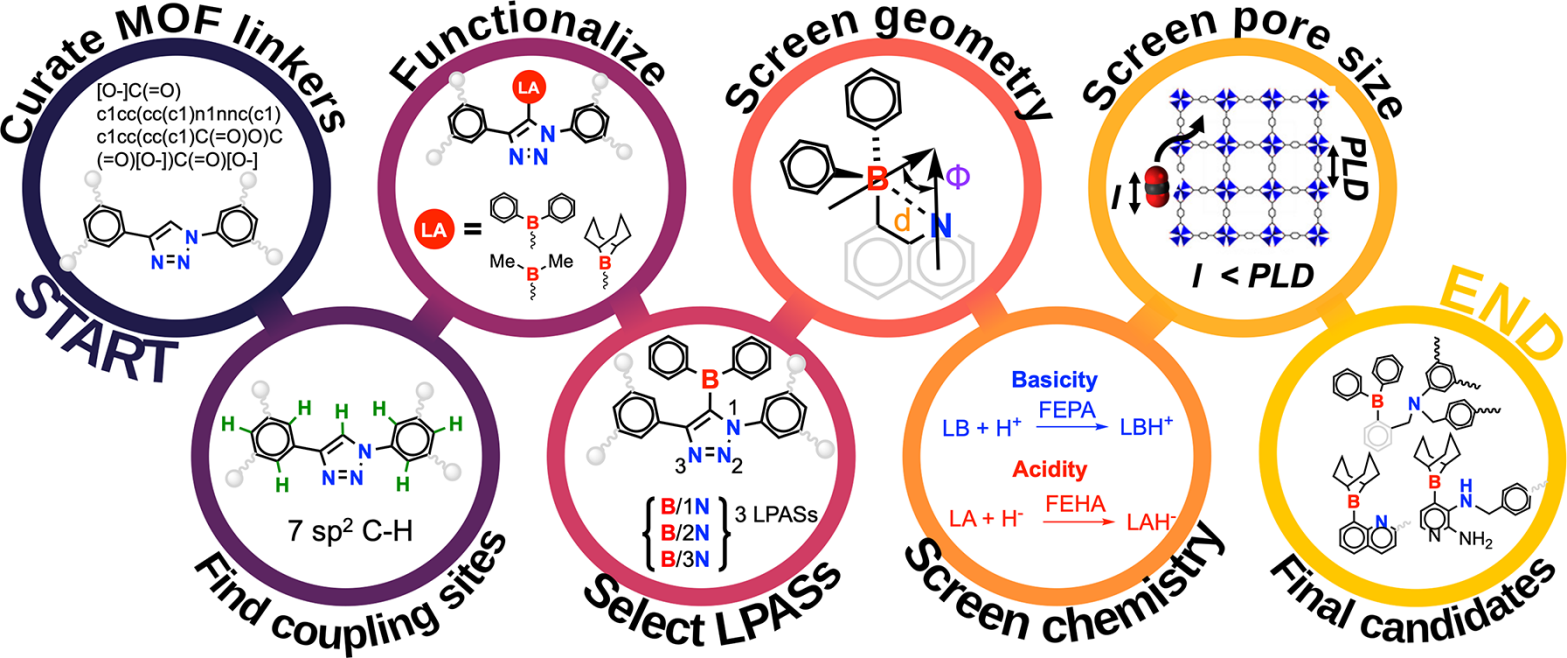
Overview of the workflow for the generation of thousands of LPASs by functionalizing 1043 SLs curated from the CoRE2019 MOF database, followed by geometry- and chemistry-based screening to identify optimal candidates for CHTF.

**Scheme 1:**
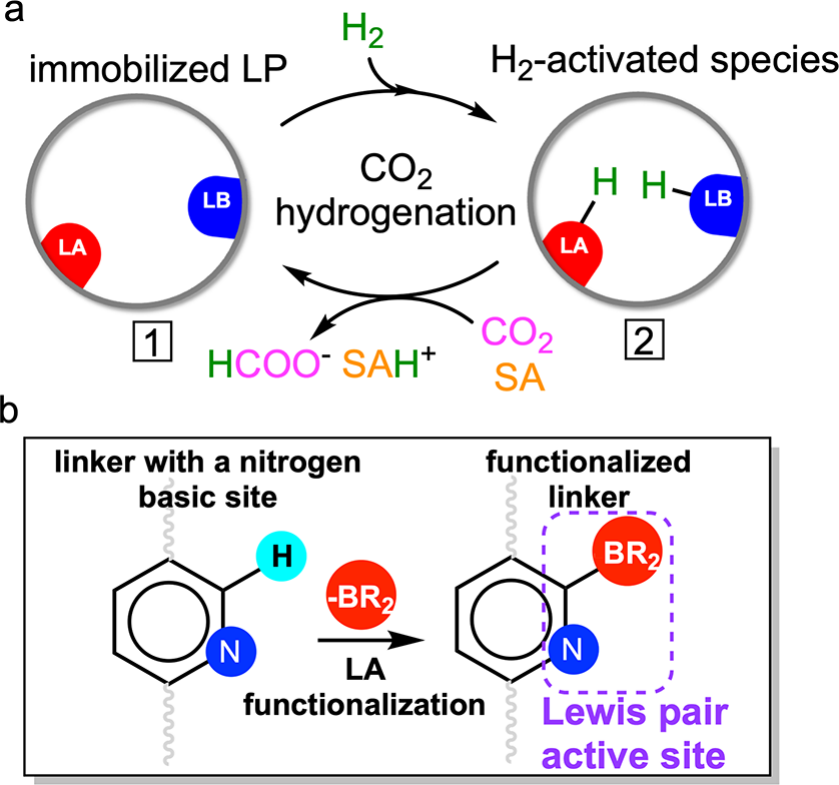
(a) Simplified Description of the Immobilized FLP-Catalyzed Direct Hydrogenation of CO_2_ to Formate; SA Represents the Sacrificial Agent Used to Drive Product Release; (b) General Strategy to Create FLP Active Sites in Porous Materials via the Functionalization of Existing Nitrogen-Containing Organic Building Blocks

We selected the CoRE2019 database, which contains a comprehensive collection of synthesized MOFs resulting from many years of experimental work, as the starting point for our study.^34^ We begin with the top-down curation of 1043 organic linkers (seed linkers or SLs) from this database that have at least one exploitable nitrogen atom as a basic site in an FLP environment (see Section S1 for details of the linker curation). After the initial refinement of these linker geometries at a semiempirical level of the theory (GFN2-xTB^35^), a rigorous in-house Python-based protocol was applied, in a bottom-up fashion, to automatically functionalize each of these linkers in all coupling sites with an acidic borane (-BR_2_) fragment.^36^ The amenable coupling sites are defined as sp^2^ carbon atoms with available hydrogens, which were identified using a series of rules based on the connectivity of atoms in each linker geometry. This strategy is motivated by the well-established chemistry of the borylation of arenes, used in the context of FLP chemistry.^37−40^ Three –BR_2_ units [R = methyl (Me), 9-borabicyclo(3.3.1)nonyl (BBN), and phenyl (Ph)] featuring diverse steric and electronic environments were introduced as the LA fragments (Figure 1), which together with the existing nitrogen basic centers constitute the potential FLP sites in the linkers (see Scheme 1b). Each borylation product therefore constitutes a distinct, albeit synthetically feasible, Lewis-pair-functionalized linker. Since each linker has multiple coupling sites, the functionalization procedure yields 25,522 derivative linkers (DLs). Each DL structure is first optimized at the GFN2-xTB level and then subsequently analyzed to identify the plausible LPAS with the following qualifying criteria: (i) the donor–acceptor distances are within the 2.0–4.0 Å range, (ii) the B–O distances are greater than 1.9 Å, and (iii) the N centers are sp^2^- or sp^3^-hybridized. The distance-based criteria (i) and (ii) eliminate all adduct-forming Lewis pairs due to the close proximity between the B and neighboring N or O (carboxyl) groups, as well as pairs in which the donor and acceptor centers are too far from each other to induce hydrogen activation, which is the first step in the mechanism of the hydrogenation cycle.^27^ The second criterion removes all sp-hybridized nitrogens (e.g., nitriles/isonitriles) that are presumed to be too weak basic sites for this step. We note that if multiple nitrogen centers are present in the DL structure, the acid unit in principle can satisfy these criteria with more than one nitrogen site, leading to multiple LPAS environments. Overall, 17,837 LPASs were identified to be carried forward to the subsequent screening step.

To illustrate this procedure, we take linker 0010 (corresponding to Core2019 MOF IDs ACAKUM, ACALIB, etc.) as a representative example (Figure 1, first bubble). It has seven C(sp^2^)–H sites (second bubble); functionalization at each coupling site with –BPh_2_ acidic fragments yields seven DLs (and more with the other Lewis acids; third bubble). In each DL, the positioning of the –BPh_2_ fragment in the neighborhood of the triazole group leads to the possibility of multiple LPAS depending on which nitrogen serves as the hypothetical Lewis base site. Upon analyzing the optimized geometry of the DLs, only three B–N environments satisfy the criteria of an LPAS (fourth bubble). The rest of the possibilities were discarded due to their nonconformity with the distance criteria or the formation of a quenched boron center (defined by B/O or B/N distances less than 2.0 Å).

#### 2.1.2 Descriptors for the CHTF Reactivity of LPASs

**Figure 2:**
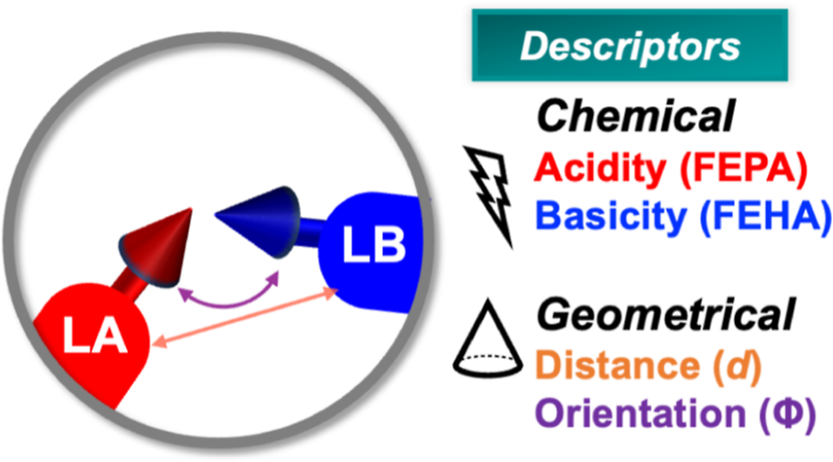
Chemical and geometrical descriptors for the CHTF activity of LPASs.

The catalytic behavior of an LPAS is determined by its chemical and geometric composition as exemplified by our recent works.^26,27^ While the former is associated with the local chemical environment of the Lewis pairs, the latter aspect refers to the spatial arrangements of the donor–acceptor centers in the active site. Thus, the next step is to devise a suitable screening procedure for identifying highly active LPAS considering both the chemistry and geometry of the candidates. Recently, some of us proposed a set of straightforward intuitive chemical and geometrical descriptors which enable a relative comparison of the CHTF activity of immobilized B–N FLPs based on minimal active site information (see Figure 2).^26,27^

##### 2.1.2.1 Geometrical Descriptors

**Figure 3:**
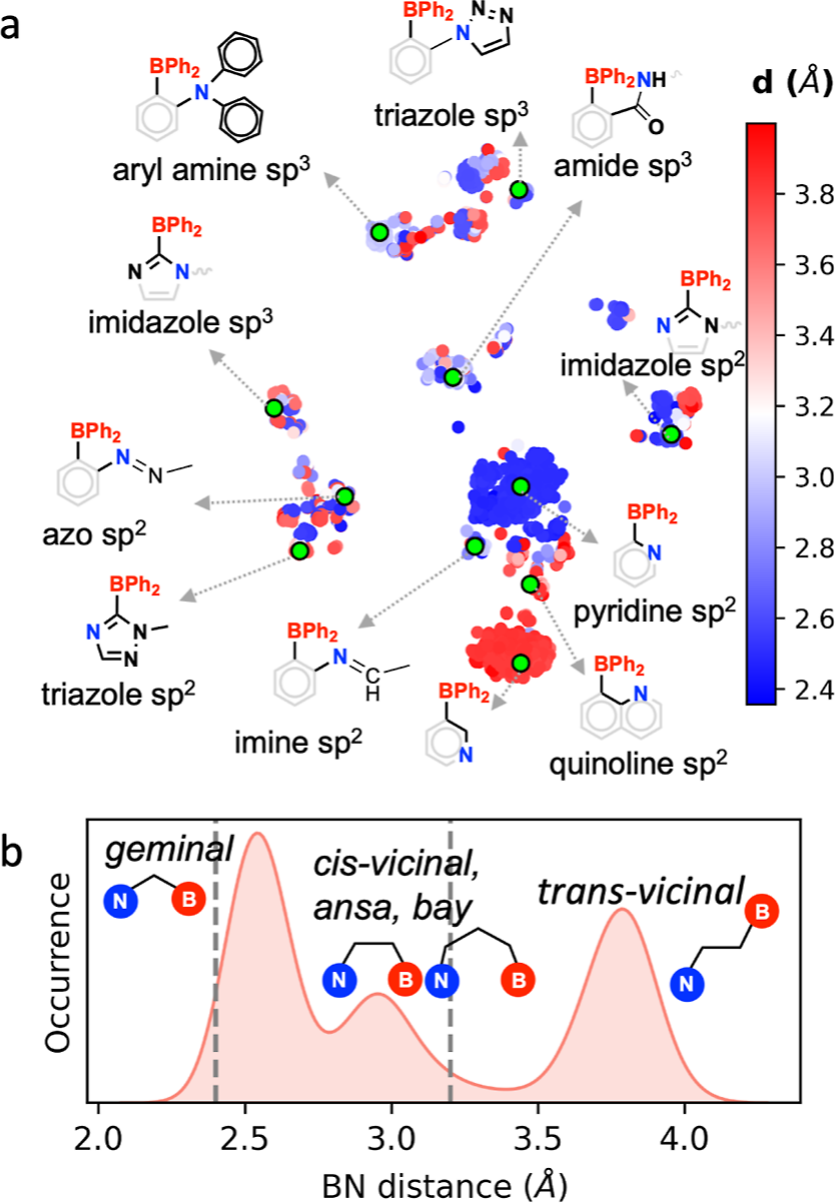
(a) Chemical diversity: 2D t-distributed stochastic neighbor embedding^44^ (t-SNE) map of the chemical diversity of all LPASs featuring –BPh_2_ as the LA fragment. The embedding was generated from the atomic spectrum of London and Axilrod–Teller–Muto potential (SLATM^45^) representation of the B and N centers (see Section S2 for details). Each point corresponds to an LPAS colored by the B–N distance. Some selected LPASs are shown, which are representative of the various clusters, along with the hybridization and local environment of the nitrogen centers. (b) Geometrical diversity: histogram of the distribution of BN distances in the optimized LPAS geometries. The gray dotted lines correspond to the range of distance that is associated with high activity for CHTF.

To measure the influence of LPAS geometry on activity, we invoke two descriptors: relative distance (*d*) and orientation (angle, Φ) of the donor–acceptor units assumed during the catalytic cycle.^27^ While *d* is trivially calculated from the distance between the B and N centers, Φ is estimated from the angle between their open coordination sites for substrate binding. In a previous study, we introduced Morse potential-based nonlinear scaling relationships to map these two geometrical descriptors to the activity of FLP catalysts for CHTF. From the resulting activity map, we found that LPASs featuring B–N distances in the 2.4–3.2 Å range (shown by dotted vertical lines in Figure 3b) and relative orientations between 70 and 140° are substantially more reactive than other geometric arrangements.^27^ Note that it is important to extract the descriptors from the intermediate **2** of the catalytic cycle to capture the effect of the reaction environment, i.e., the inclusion of the H_2_ molecule (see Scheme 1a).

##### 2.1.2.2 Chemical Descriptors

Similarly, the effect of chemical composition can be quantified from the individual intrinsic strength of the Lewis components estimated from two descriptors: free energy of hydride attachment (FEHA) and free energy of proton attachment (FEPA) to the corresponding acid and base centers, respectively. Recently, we proposed a computational framework based on linear free energy scaling relationships^41−43^ in which these chemical descriptors are mapped to CHTF activity enabling the screening of a database of intermolecular B/N and B/P Lewis pairs.^26^ One of the major findings of our study was that the acidity and basicity of the Lewis components need to be appropriately complemented to ensure high catalytic performance. In other words, the cumulative acid–base strength of the pair dictates catalytic activity regardless of the individual strength of the components. Most importantly, the efficacy of our model was confirmed by the experimental demonstration of the first reported catalytic turnovers in FLP-catalyzed hydrogenation of CO_2_.

The four descriptors described above allow the identification of regions of theoretically derived maximum CHTF activity by balancing the acidity and basicity of the Lewis components and by controlling their spatial arrangements.

#### 2.1.3 Diversity of LPASs

The chemical and geometrical diversity of the collection of LPASs with –BPh_2_ as the acid fragment are illustrated in Figure 3. Figure 3a shows the two-dimensional representation of the chemical diversity using a two-dimensional t-SNE^44^ map based on the atomic SLATM^45^ representations of the donor (N) and acceptor (B) centers. The vertical axis roughly corresponds to the hybridization of the nitrogen center in the LPAS: sp^3^ N sites occupy the upper half of the plot while sp^2^ N sites appear at the bottom half (see Figure S8). The horizontal axis captures the variation of the structural environments in which the nitrogen center is found (e.g., the presence of heteroatoms in the heterocyclic system). The clustering of the different FLP environments is largely based on the distance between the B–N centers as illustrated by some representative systems in Figure 3a. The geometric diversity is shown by the 1D histogram of the B–N distances in the optimized LPAS geometries (Figure 3b). Three major peaks around 2.5, 3.0, and 3.8 Å in the distance histogram correspond to geminal, *cis*-vicinal/*ansa*/bay, and *trans*-vicinal type spatial arrangements of the Lewis sites, respectively.

### 2.2 Screening of LPASs

#### 2.2.1 Geometrical Criteria

The optimized structures of the curated LPASs are first subjected to a geometry-based screening to recognize the candidates that feature appropriate spatial arrangements in the active sites for the activation of H_2_ and subsequent hydride transfer to CO_2_.^27^ This is primarily due to the stringent nature of the geometric criteria (particularly dominated by Φ) that allow a preliminary screening of the LPAS library and convenient estimation of the relevant descriptors.^27^ The geometry of the intermediate **2** for each LPAS was constructed in an automated fashion.^46^ The GFN2-xTB-optimized geometries were then filtered to remove fragmented structures, and the geometric descriptors *d* and Φ were determined.^46^ The vast majority of the LPASs were ruled out by this criterion, leading to a significant reduction of the candidate space. Many of the disqualified candidates feature geminally disposed B and N centers (typical *d* around 2.5 Å); while they satisfy the distance criteria, their relative orientations deem them unsuitable for CHTF, as such closely placed Lewis centers lead to small Φ values. Such orientations are unfavorable for both kinetics of the H_2_ activation steps and the thermodynamics of the product release step, both of which have been previously associated with poor CHTF activity.^27^ A comparison of the distribution of the Φ values (see Figure S5) reveals that the steric environment of the LA fragment has an impact on determining the orientation, particularly in the prescribed range of high activity; more than 87% of the qualified LPASs contain the larger substituents –BPh_2_ and –BBN. The geometries of the candidates satisfying the geometry-based criteria (around 400) were further refined at the PBE0-D3BJ/def2SVP level and the final *d* and Φ values were collected. These candidates were then carried over for the subsequent chemistry-based screening step.

To estimate the effect of including the linker within the actual MOF structure, a functionalized derivative of the widely used azobenzene-4,4′-dicarboxylic acid (ABDC) linker, termed here 2-BBN-ABDC, was employed to construct a MOF with the primitive cubic (pcu) topology containing a widely used metal node, Zn_4_O (Figure S2). Upon geometry optimization of the MOF at the PBE-D3 level of theory, the geometric descriptors from the linker were extracted (see Supporting Information for details). Crucially, these descriptors remain similar to those obtained in the molecular model, implying that the parameters derived from the linkers can be extended to realistic MOF structures.

#### 2.2.2 Chemical Criteria

**Figure 4:**
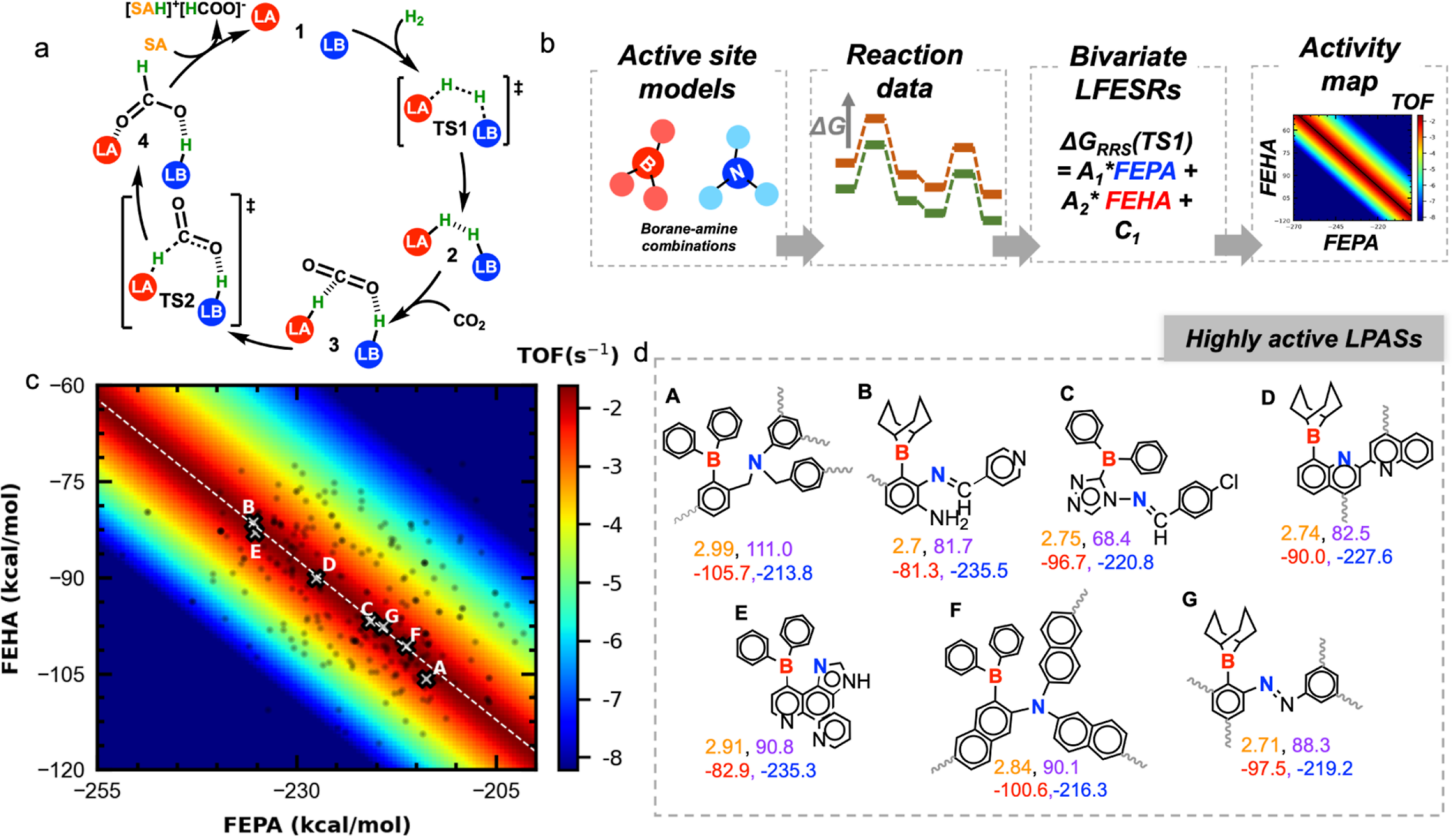
(a) Catalytic cycle for the FLP-catalyzed direct hydrogenation of CO_2_ to formate. LA = Lewis acid; LB = Lewis base. Off-cycle resting states such as an FLP-CO_2_ adduct or a quenched LA-LB dative adduct are omitted for the sake of generality. (b) Schematic depiction of the steps involved in the construction of the activity map. (c) Activity map describing the TOF for the CHTF cycle as a function of FEPA and FEHA for B/N FLPs. Each point represents an LPAS selected from the geometry-based screening step. The TOF is presented in a logarithmic scale. The white dotted line across the activity map depicts the desired complementarity between FEPA and FEHA to achieve maximum TOF. (d) The chemical and geometric composition of a selection of prospective LPAS candidates (represented by gray crosses) for CHTF along with four descriptor values (*d*:orange, Φ:violet, FEHA:red, FEPA:blue). The gray curvy lines represent carboxyl groups through which the linker is connected to the metal-containing nodes in the MOF structure.

Having identified the LPASs with the appropriate spatial arrangement in the active site, we now search for candidates whose complementary Lewis acidity and basicity are optimal for the reaction in question. The balance between the intrinsic reactivity of the components is visualized in the map presented in Figure 4. Here, by establishing linear free energy scaling relationships, the theoretically derived TOF (plotted along the color axis) is described as a function of FEPA and FEHA (plotted along the *x* and *y* axes, respectively). These scaling relations were established by analyzing free energy profiles computed for a selected pool of B/N FLP combinations based on the previously established mechanism of hydrogenation (see Figure 4a) that involves heterolytic H_2_ cleavage by the FLP, binding of the CO_2_ substrate, transfer of the hydride and proton for the reduction, and release of the formate product.^26,27,32,47−51^ A schematic depiction of the steps involved in the construction of the activity map is shown in Figure 4b and further details are given in the Supporting Information (see Section S3) and elsewhere.^52^ The maximum activity region in Figure 4c, implying the desired complementarity between acidity and basicity, is indicated by the dark red patch across the map. The candidates that appear in this region are anticipated to have high activity since they satisfy both the geometry and chemistry criteria. Upon moving away from the red region to either side, the activity diminishes due to the increasingly difficult product release or higher activation barrier for H_2_ cleavage/hydride transfer (Figure S3).^26^ The relative activity of the candidates is compared by placing them on the activity map according to their descriptor values. The sign convention used here defines a more negative FEHA/FEPA value as having a stronger acceptor/donor ability. A broad distribution of FEPA values spanning from −200 to −250 kcal/mol is observed, indicating the diversity of the basic sites present in this subset. Interestingly, for LPASs with a particular LA fragment, the FEHA distribution remains fairly broad (e.g., −80 to −116 kcal/mol for BPh_2_) although comparatively narrower than the range of FEPA values. This implies that the third (aryl) substituent on the boron center, the linker backbone itself, has a strong influence on Lewis acidity of the boron. The reference chemical descriptor values of a few common experimentally used LAs and LBs are provided in Supporting Information (Table S2).

Analysis of the composition of the subset of LPASs that satisfy both sets of criteria reveals certain key chemical and geometrical patterns leading to high activity. By examining some of the representative candidates (Figure 4d, **A**–**G**), it is revealed that stronger LA units (FEHA < −100 kcal/mol) are best paired with weak basic sites such as aryl amines or azo nitrogens, see, for example, **A** which features a triaryl borane unit (FEHA = −105.7 kcal/mol) combined with a triaryl amine basic motif (FEPA = −216.2 kcal/mol). Conversely, diminished acidity on the boron sites requires stronger complementary basic sites; in such “reverse” scenarios, aliphatic, imidazole, and imine basic sites are more appropriate, as in **B** which features a much weaker BBN-containing acid unit (FEHA = −81.3 kcal/mol) paired to a stronger imine basic unit (FEPA = −235.5 kcal/mol). Finally, LPASs containing acid sites with moderate FEHA values feature basic partners of intermediate strengths (**C** and **D**). However, a general observation in all of these highly ranked LPASs is that both the B and N centers are somewhat rigidified either by being embedded into a ring system (endocyclic) or by being surrounded by three aryl groups so that the donor and the acceptor centers are locked into a distance and orientation appropriate for the reaction of interest. The most frequently observed compositions involve locking in a *cis*-vicinal arrangement (*d* and Φ of 2.8 Å and 80–90°, respectively), in which the B and N centers are tethered to each other by a two-atom bridge from a six/five-membered aromatic core. Slightly higher separations (2.9 and 3.0 Å in **A** and **E**, respectively) between the Lewis centers are observed in the *ansa* or *bay*-type arrangements, where the nitrogen centers are often part of imidazole or pyrazole cores. The highest angle is observed in **A** (111.0°) due to the *ansa*-type arrangement in which the sp^3^ nitrogen takes a benzylic position with respect to the phenyl ring that contains the acid unit. Note that some of these arrangements (vicinal in particular, such as **B**, **C**, **F**, **G**) identified from our screening protocol have structural similarities to the intramolecular FLP system which is known for experimental demonstration of CO_2_ hydrogenation albeit without the catalytic turnover (see Section S7).^47^

#### 2.2.3 Beyond the Active Site: Pore Size-Based Criteria

**Figure 5:**
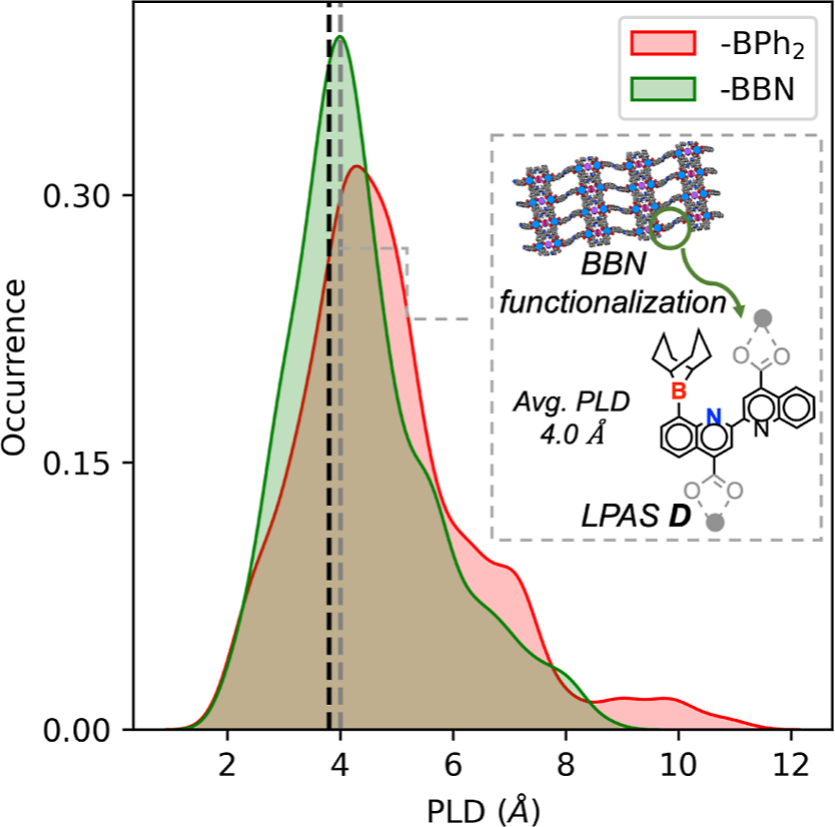
Histograms showing the distribution of the PLDs derived from the combinations of 54 BPh_2_ and 27 BBN-containing linkers combined with 53 metals. The black line corresponds to the smallest usable PLD of 3.8 Å, which corresponds to the kinetic diameter of the CO_2_ molecule 3.3 Å plus the mean absolute error of the prediction model 0.46 Å. The gray dotted line corresponds to the mean PLD of a promising representative linker, estimated by taking an average of over 53 PLD values resulting from combinations with 53 metals (inset).

Because these LPASs are engineered to be incorporated into a porous environment, one crucial factor that could limit their activity is the ability of the substrate molecules to access the FLP active sites. Therefore, as a final screening step, we examine the compatibility between the pore size of the potential MOF catalysts derived from these highly active LPASs and the kinetic diameter of the substrate molecules. To this end, we trained a regression model by exploiting the machine learning framework and CSD MOF data from Rosseinsky et al.^53^ to predict the pore limiting diameter (PLD), a quantitative metric of MOF porosity, from the identity of the metal atom and the linker SMILES alone. Details of the regression model training and cross-validation are given in the Supporting Information. We use this model to readily estimate the PLDs of the MOFs derived from the combinations between the LPASs satisfying the chemical and the geometrical criteria and the 53 metals present in the database. To achieve a functional LPAS within a porous environment, the PLD must be greater than 3.8 Å (the kinetic diameter of the CO_2_ molecule, which is 3.3 Å plus the mean absolute error of the regression model, 0.46 Å).^54^Figure 5 shows the distribution of the PLDs for 4293 metal-linker combinations (54 BPh_2_– + 27 BBN-containing LPASs combined with 53 metals).^55^ Our results reveal that the majority of those combinations are compatible with CHTF since their PLD is larger than 3.8 Å (indicated by the black line in Figure 5). Based on this PLD threshold, 28 out of 54 BPh_2_-containing and 19 out of 27 BBN-containing linkers are prioritized. Note that this PLD-prediction model offers a simple and quick guide to prioritize the LPAS candidates most likely to generate MOF structures that can accommodate the substrate molecules. This has been further validated by constructing full MOF structures for a few representative ditopic, tritopic, and tetratopic LPAS-containing linkers combined with appropriate metal nodes in various compatible topologies (see Section S6 and Figures S11 and S12 in Supporting Information). The computed pore properties from the resulting MOF structures fully support the selection based on the prediction from the regression model.

For one representative linker featuring the LPAS D (see inset of Figure 5 for structure), with the BBN group as the LA and pyridine nitrogen as the LB component, we explicitly calculate the free energy profiles (reaction intermediates and transition states) for CHTF. To examine the effect of the MOF environment, the geometry of the corresponding seed linker was harvested from the CoRE MOF database (CSD refcode: ASECUY), and the terminal bridging oxygen atoms (gray in Figure 5) were constrained during the geometry optimization of the reaction intermediates and transition states. The computed energy span is consistent with the high activity predicted from the screening, highlighting the minimal effect of the pore environment, provided it is spacious enough to accommodate the substrate molecules (see Figure S9).

### 2.3 Guidelines for Designing Highly Active LPASs for CHTF

**Scheme 2:**
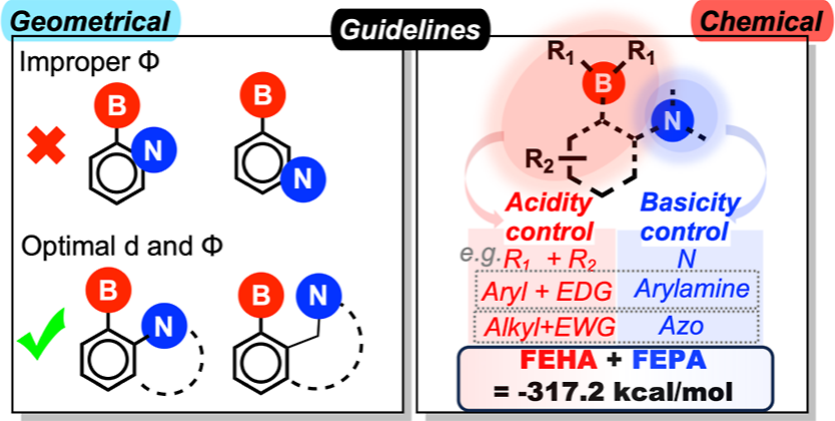
Geometrical and Chemical Design Rules for Constructing Highly Active LPAS for CHTF; EWG/EDG: Electron-Withdrawing/Donating Groups

Analyses of the screened candidates lead to the following design principles for the construction of highly active LPASs for CHTF (see Scheme 2). Given a particular basic site that is either embedded within an aromatic core or attached to one, the boron-based acidic site is most effective if it is separated from it by not more than three atoms; otherwise, it will hold the donor and acceptor centers at too great a distance (>4.0 Å) to show any cooperative reactivity (especially for the initial hydrogen activation step). On the other hand, geminal positions should also be avoided, as they place the Lewis centers at such short distances that the reaction cavity is not spacious enough (implied by low Φ values) to maintain the proper orientation of the H_2_ molecule during bond cleavage.

The optimal spatial arrangements are the *cis*-*vicinal*, *ansa*, and *bay*-type dispositions that offer the appropriate B/N separation and orientation between the acid–base sites required for high catalytic performance. Large acid substituents are generally advisable to maintain the proper orientation between the centers and to avoid quenching with the basic centers. Regarding the chemical composition of the LPAS, the acidity of the boron fragments must be adjusted according to the basicity of the nitrogen site to preserve the cumulative acid–base strength required for high CHTF activity and, unlike the geometry-based requirements, this can be achieved with many different types of acid–base combinations. While choosing the acid fragment, two factors must be considered: (a) the strength of the nitrogen basic site and (b) the electronic nature of the aromatic core to which it is introduced. For instance, for a weak basic site and an electron-rich core, two aryl substituents on the boron centers provide the complementary Lewis acidity, such as in **F** (see Figure 4d). This might not be necessary if the core is electron deficient such as in **G**, where a –BBN unit is sufficiently acidic to complement the weak azo basic site. While we have identified several such chemically balanced LPAS compositions here, many more could be envisioned simply considering the delicate interplay between the factors discussed above. Nevertheless, cumulative strength for any new Lewis pair combination can be readily estimated by computing the corresponding acidity and basicity descriptors using straightforward DFT computations.^26^ The desired balance between the acidity and basicity leading to maximum TOF is given by the criterion that FEHA + FEPA = −317.2 kcal/mol (represented by the white dotted line across the activity map in Figure 4c) within the range of explored chemical descriptor values. This can be used as a rule-of-thumb for the appropriate chemical design of the LPASs. We note that since these guidelines are derived from a local descriptor-based approach (extracted only from the Lewis components of the active site), we expect them not to be limited to MOF linkers but also transferable to a broad family of materials that exploit structurally well-defined organic building blocks. Other bottom-up porous materials, such as covalent organic frameworks and porous aromatic frameworks, may serve as similar templates provided they possess the appropriate porosity to allow the access of the reactant molecules to the active site.

## 3 Conclusions

In this work, we formulate simple guidelines for designing chemically and geometrically appropriate immobilized frustrated LPASs that ensure high activity for the direct catalytic hydrogenation of CO_2_ to formate. By introducing complementary acidic fragments in the vicinity of intrinsically present nitrogen basic sites present in the organic linkers of MOFs, we construct a sterically and electronically diverse library containing several thousands of possible catalytically active sites. The relative hydrogenation activity of the candidates in the library is determined by estimating a set of straightforward and intuitive descriptors representing the basicity and acidity of the donor and acceptor centers, respectively, along with their separation distance and orientation relative to one another. As these active sites will be employed in the context of porous environments, the accessibility of the active site for the CO_2_ substrate was also considered in the screening workflow via the machine-learning-predicted pore-limiting diameter. This enables the identification of key chemical and geometrical features responsible for high catalytic activity. Based on these results, we propose that spatial donor–acceptor arrangements in the *cis*-*vicinal*, *ansa*, or bay positions maintain the appropriate distance and orientation required for high activity. We expect that different types of acid–base combinations, resulting from the interplay between the electronic nature of the acid fragments, base fragments, and the associated linker cores, will impart on the engineered active site the cumulative acid–base strength desired for high activity. Several such FLP environments are identified and may be incorporated in a broad range of materials (e.g., covalent organic frameworks, coordination polymers, or porous aromatic frameworks). These results lay the groundwork for further exploration of immobilized active sites for important catalytic transformations.

## Data Availability

All data are made available as a Materials Cloud repository: https://doi.org/10.24435/materialscloud:90-b6. The *crosscoupler* tool used to functionalize the linkers in the seed database is available on GitHub at https://github.com/lcmd-epfl/FORMED_ML.

## Supporting Information Available

Data set generation and curation; computational details; construction of the plots for mapping chemical composition to activity; conformational analysis of the selected linkers; distribution of the geometric descriptors for all LPASs; distribution of the chemical descriptors for LPASs with appropriate geometry; free energy profile of a selected LPAS for hydrogenation; machine-learning model for porosity prediction; structural analysis of MOFs with different topologies; and comparison with the experimentally reported intramolecular FLPs for stoichiometric CO_2_ hydrogenation (PDF)
