# Supplementary material for: Engineering Frustrated Lewis Pair Active Sites in Porous Organic Scaffolds for Catalytic CO2 Hydrogenation
Source: J Am Chem Soc. 2024 May 30;146(23):15806–14. doi: 10.1021/jacs.4c01890 (PMC11177311; doi:10.1021/jacs.4c01890)
Supplement: Supplementary file 1 — ja4c01890_si_001.pdf [file ja4c01890_si_001.pdf]

Supporting Information for Engineering Frustrated Lewis Pair Active Sites in Porous Organic Scaffolds for Catalytic CO_2 Hydrogenation

Shubhajit Das,[†,‡] Ruben Laplaza,[†,¶] J. Terence Blaskovits,[†,§] and Clémence Corminboeuf*[,†,¶]

†*Laboratory for Computational Molecular Design, Institute of Chemical Sciences and Engineering, École Polytechnique Fédérale de Lausanne (EPFL), 1015 Lausanne, Switzerland*

‡*Present Address: Faculty of Chemistry and Food Chemistry,Technische Universität Dresden,01062 Dresden, Germany*

¶*National Center for Competence in Research-Catalysis (NCCR-Catalysis), École Polytechnique Fédérale de Lausanne, 1015 Lausanne, Switzerland*

§*Present address: Max-Planck Institute for Polymer Research, Ackermannweg 10, 55128 Mainz, Germany*

E-mail: clemence.corminboeuf@epfl.ch

S1: Construction of the library of LPAS

Initial linker curation: We begin by performing a benzene substructure (as a SMILES string) search of the CoRE2019 database through the web-mofiD interface, which returned 7285 MOF entries. Each entry correspond to a MOFid represented in a specialized SMILES format where the chemical components are separated by a "dot". The initial seed database of linkers was curated using the following protocol. First, SMILES strings corresponding to the organic linkers were separated from the MOFid entries. The linkers containing the anionic carboxylic groups were neutralized by adding protons using RDKit. Only the linkers with at least one nitrogen atom were retained and duplicates were removed. 3D structures were generated from the SMILES strings using openbabel[1] and their molecular geometries were optimized at the GFN2-xTB level.[2] The optimized linker geometries were analyzed and linkers with amibiguous/improper geometries or missing hydrogens were removed at this stage. This procedure yielded 1043 nitrogen-containing linkers, which we define as the "seed" database.

Functionalization and LPAS selection: The functionalization of the 1043 linkers from the seed database yielded 9111, 9345, and 7066 (25,522 total) derivative linkers (DLs) for -BMe_2, -BPh_2 and -BBN, respectively. Note that the number of DLs for a -BR_2 unit depends on the mutual steric compatibility of the coupling site and of the acid substituent, hence the different number of DLs for each LA component. The molecular geometries of the DLs were then optimized at the GFN2-xTB level and possible LPAS environments were selected according to the criteria provided in the main text. This procedure yielded 6246, 6400, and 5191 (17837 total) LPASs for -BMe_2, -BPh_2 and -BBN substituents, respectively.

S2: Computational details

Calculation of the geometrical and chemical descriptors

The initial geometries of the intermediate **2** corresponding to all LPASs with d < 3.8 Å were constructed in an automated fashion by appending a proton and a hydride to the corresponding N and B centers, respectively. The geometric descriptors, relative distance (d) and relative orientation (Φ) of the LA and LB units were first extracted from the optimized geometry of the intermediate **2** at the GFN2-xTB level. d is calculated as the distance

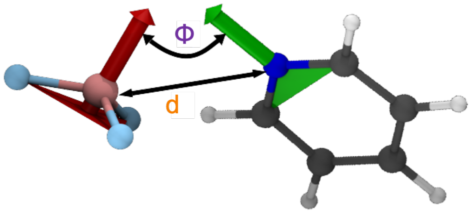

Figure S1. Schematic depiction of the detailed definition of the two geometric descriptors used to monitor the effect of geometric constraint on catalytic activity.

between the N and B atoms of the LB and LA motifs, respectively, in an LPAS. Φ is defined as the angle between two vectors defining the LA and LB fragments. The mechanistic significance of both descriptors is discussed in previous work.[3] The LA vector passes through the centroid of the three boron substituents and the boron atom. For an sp^2 nitrogen, the LB vector is constructed from the centroid of the nitrogen atom and its two adjacent atoms, and passes through the nitrogen atom. For an sp^3 nitrogen, the procedure is the same as the determination of the LA vector. The selected LPAS geometries with 2.4 Å < d < 3.2 Å and 70°< Φ < 140° were further reoptimized at the PBE0-D3BJ/def2SVP[4–8] level of theory and geometric descriptors were extracted.

The chemical descriptors, FEPA and FEHA, were estimated from the free energy change associated with following reactions, as defined by equation (1) and (2),

$$\boxed{[\boldsymbol{N}] + \boldsymbol{H}^{+} \xrightarrow{\text{FEPA}} [\boldsymbol{NH}]^{+}} \quad (1)$$

$$\boxed{[\boldsymbol{B}] + \boldsymbol{H}^{-} \xrightarrow{\text{FEHA}} [\boldsymbol{BH}]^{-}} \tag{2}$$

where [B] and [N] correspond to acid and base fragments in an LPAS. According to the sign convention used here, a more negative FEPA/FEHA value corresponds to a stronger donor/acceptor ability. The molecular geometries of the protonated, hydride-attached, and neutral LPAS were optimized at the PBE0-D3BJ/def2-SVP level of theory as implemented in Gaussian 16 software.[9] To refine the electronic energies, further single-point computations were performed at the PBE0-D3BJ/def2-TZVP level.[8] To include the effect of entropic contributions, free energy corrections considering only vibrational contributions (corrected using the rigid-rotor approximation as implemented in the GoodVibes program[10]) were added to the single-point energies at the PBE0-D3BJ/def2-TZVP level of theory. Translational and rotational contributions were omitted as they are not well-defined for immobilized molecular systems. All calculations were performed in the gas phase.

The t-distributed stochastic neighbor embedding (t-SNE) map was constructed from the concatenated spectrum of London and Axilrod-Teller-Muto potentials[11] (SLATMs) of the B and N sites in the LPAS geometries. The SLATM representation contains one, two- and three-body potentials bagged over pairs and triads of element types.

From the promising LPAS-containing linkers, we selected seven representative ones (featuring two, three, and four connection points) to build the corresponding MOF structures combined with six selected metal nodes. These building blocks were combined using PORMAKE.[12] During the MOF construction, the choice of topologies was restricted to the top 100 options selected based on their occurrence in the DigiMOF database.[13] Furthermore, the number of atoms per unit cell were restricted to 2000 while a limiting threshold of 60 Å was applied to the cell parameters to reduce computational expense. All generated structures were subsequently optimized using the Universal Forcefield for Metal-Organic Frameworks (UFF4MOF) with GULP.[14,15] The geometry optimizations of the MOFs were performed in two stages: keeping the cell fixed, the atomic positions were relaxed first followed by a full

relaxation of the cell until a convergence threshold of 0.02 eV/Å was reached. The optimized MOF structures were used for subsequent structural analysis. The Zeo++[16] program was used to compute the corresponding geometric properties, including PLD. An in-house python script was used to measure the distance between the LPASs within the pore.

Comparison of the geometrical descriptors with a periodic model

To realize the effect of including the linker within the actual MOF structure, a BBN-functionalized azobenzene-4,4'-dicarboxylic acid (ABDC) linker (#91 in the seed database, termed here 2-BBN-ABDC) was chosen and a MOF was constructed using a widely-used metal node, Zn_4O, with the primitive cubic (pcu) lattice topology. The MOF construction was performed with the Autografs framework builder as implemented in ADF2018.01 software.[17] The geometry of the MOF was optimized using the GPW formalism as implemented in the QUICKSTEP module of the CP2K 7.1[18] package. PBE-D3BJ exchange-correlation[7,19] assessment is used along with Goedecker-Teter-Hutter (GTH) pseudopotentials[20] and triple-zeta valence polarized basis sets for all atoms except Zn, for which a double-zeta valence polarized basis set was used.[21] The geometric descriptors (d ,Φ) extracted from the three linkers present in the primitive unit cell of the optimized MOF structures are (2.84 Å, 89.9°), (2.93 Å, 85.3°) and (2.85 Å, 87.9°). This suggests that the descriptors are minorly affected within the MOF geometry compared to the same LPAS in the molecular environment (2.77 Å, 80.0°).

In order to assess how the functionalized borane units might be accommodated within the MOF pores, we computed the steric volume occupied by the different components in the above-mentioned MOF structure. For this purpose, we used the libarvo library and tabulated van der Waals radii.[22] The optimized unit cell has a volume of 8767.3 $\AA^3$, out of which 250.0 $\AA^3$ are occupied by the nodes, 3 $\times$ 306.0$\AA^3$ are occupied by the linkers, and 7601.7 $\AA^3$ remain free. In total, the occupied steric volume represents only a 15.4 % of the unit cell. We note that the BBN group occupies only 142.6 $\AA^3$ out of the 306.0 $\AA^3$ of each linker. Thus, the size of the total BBN functionalization remains negligible with respect to

the total pore size.

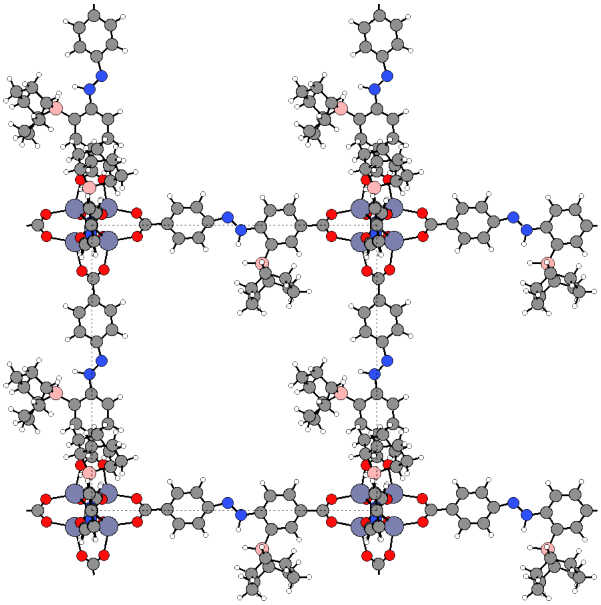

Figure S2. Geometry optimized structure of the MOF constructed with Zn_4O node and the 2-BBN-ABDC linker (#91) in pcu topology. The dashed box indicates the primitive unit cell.

S3: Mapping chemical composition to activity

Figure S3 illustrates the generalized catalytic cycle for direct FLP-catalyzed CO_2 hydrogenation to formate as described in previous experimental and computational studies.[3,23–29] The mechanism begins with the heterolytic cleavage of H_2 by the Lewis pair **1**, leading to the formation of the ion-pair **2** through **TS1**. Binding of CO_2 results in intermediate **3**, which upon hydride transfer (HT) via **TS2** leads to the formation of **4**, featuring the formate bound to the LA and LB units. The catalytic cycle is closed upon extraction of the formate by a sacrificial reagent (*e.g.* additional base or boron species) and regeneration of the catalyst. Here, a pyridine LB molecule is used as the sacrificial agent.

The chemical compositions of the LPASs are mapped to the CO_2 hydrogenation to formate (CTFH) activity by constructing an activity map which describes the turnover frequency (TOF, a metric for catalyst performance) as a function of the acidity (FEHA) and

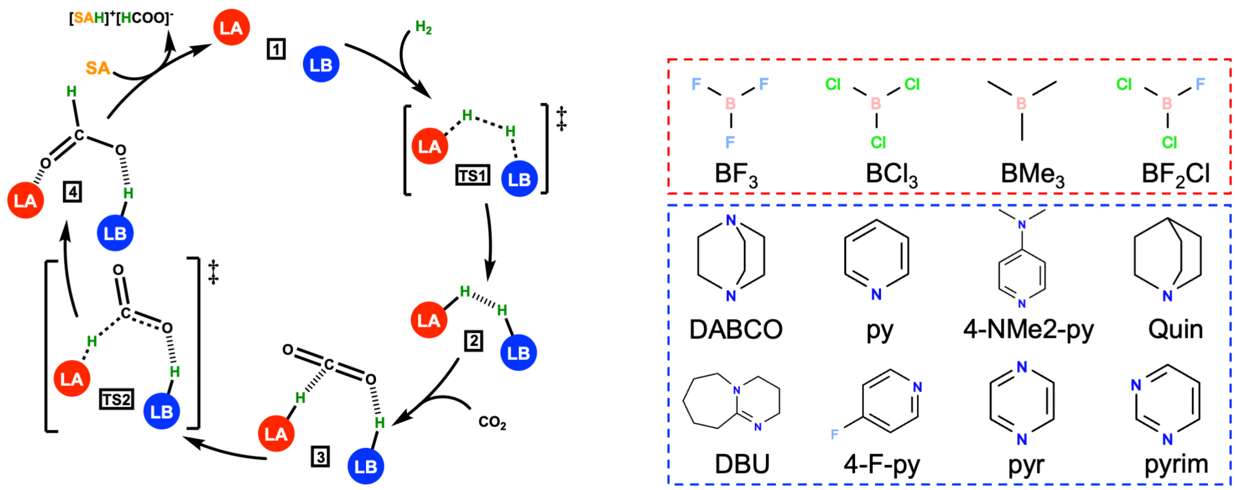

Figure S3. (Left) Catalytic cycle for the FLP-catalyzed direct hydrogenation of CO_2 to formate. LA = Lewis acid; LB = Lewis base. Off-cycle resting states such as an FLP-CO_2 adduct or a quenched LA-LB dative adduct are omitted for the sake of generality. (Right) Library of LA and LB components to build the activity map using the chemical descriptors.

basicity (FEPA) of the of the Lewis components. The general procedure for constructing activity maps can be found elsewhere.[30–33] However, in this section, we briefly describe the procedure for building the activity map described in Figure 3. The prerequisite to creating an activity map is to establish (linear) free energy scaling relationships that correlate with the relative stabilities ($\mathbf{\Delta G_{RRS}}$) of the intermediates and the transition states in the catalytic cycle and two (or several) descriptor variables. To this end, standard DFT computations were performed to estimate $\mathbf{\Delta G_{RRS}}$ of all reaction intermediates and transition states (TSs) involved in the catalytic cycle for a library of intermolecular FLPs. The library consists of four borane LAs combined with eight nitrogen LBs furnishing a total of 32 Lewis pair combinations (see Figure S3).

The $\mathbf{\Delta G_{RRS}}$ corresponding to all intermediates and transition states in the CTFH cycle are described as linear functions of FEPA and FEHA using a bivariate linear regression. Thus, ΔG_{RRS} values take the form $\Delta G_{RRS}(\mathbf{Int/TS\ n}) = A_1 * \mathbf{FEPA} + A_2 * \mathbf{FEHA} + b$, where A_1, A_2 are the slopes and b is the intercept. The activity map is constructed by determining a 2D grid spanning the entire range of FEPA and FEHA, and subsequently computing first the hypothetical free energy profile followed by the corresponding TOF

for each grid point. Plotting the descriptors FEPA and FEHA along the x- and y-axes, respectively, and the corresponding TOF values (in log scale) along the color axis yields the activity map in Figure 3.

Table S1: Coefficients obtained from the bivariate linear regression model along with the regression diagnostics correlation coefficient (R^2), and mean absolute error (MAE, in kcal/mol).

Species	A_1	A_2	b	R^2	MAE
**TS1**	0.19	0.18	61.86	0.81	1.65
**2**	0.51	0.58	163.97	0.89	2.95
**3**	0.38	0.20	87.24	0.63	2.90
**TS2**	0.61	0.58	191.264	0.88	2.70
**4**	0.42	0.60	117.96	0.93	2.47

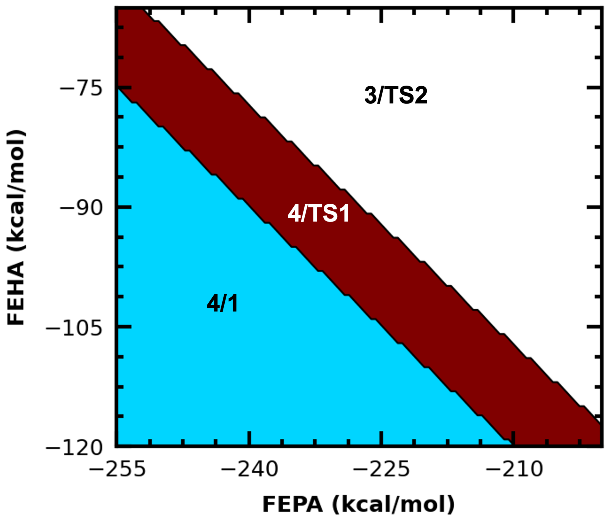

Figure S4: Map in which each grid point is colored according to the identity of the turnover-determining intermediates and turnover-determining transition states in the CTFH cycle.

S4: Conformational analysis

To estimate how the conformational space of the linkers impacts the geometric descriptors, we performed a conformational analysis on the intermediate **2** for a few representative linkers. The sampling was performed using the conformer-rotamer ensemble sampling (CREST)

program with the semiempirical quantum chemistry method GFN2-xTB.[2,34,35] The length of the metadynamics simulations was kept to the default value and the temperature was set to 298.15 K. RMSD and energy thresholds were set to 0.5 Å and 0.05 kcal/mol respectively, and an energy window of 5 kcal/mol was used throughout. Terminal atoms of the linkers were kept constrained during the simulations with a constraint strength of 1 a.u./Åto simulate the effect of the MOF environment. From the resulting ensembles, five different structures were selected (based on agglomerative RMSD clustering) and reoptimized (without any constraint) at the PBE0-D3BJ/def2SVP level of theory and geometric descriptors were calculated. The resulting standard deviations in the geometric descriptors for various conformers are 0.02 Å, 0.07 Å, 0.01 Å, and 0.01 Å in distances and 2.34°, 0.45°, 3.72°, and 5.41° in angles for candidates **A**, **D**, **F**, and **B**, respectively, which keeps them within the prescribed range of high CTFH activity.

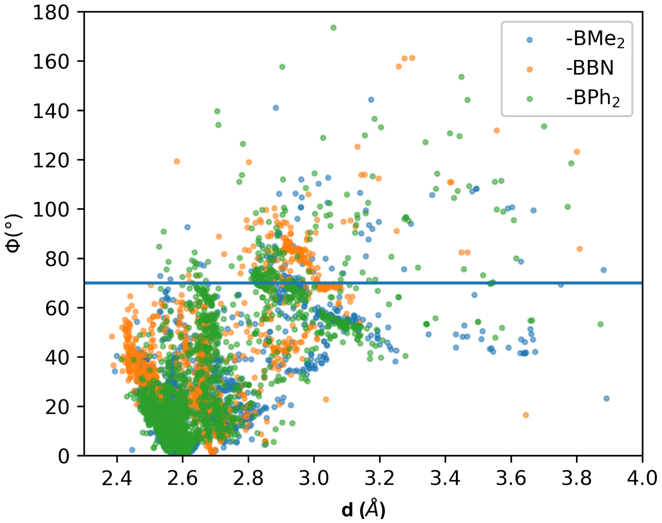

Figure S5. Distribution of the geometric descriptors for all LPASs.

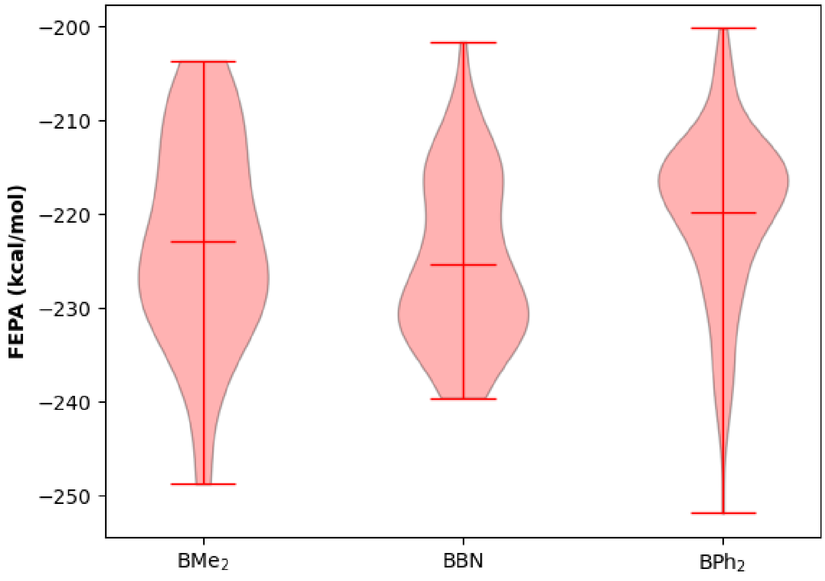

Figure S6. Distribution of the FEPA values for LPASs during screening based on chemical criteria.

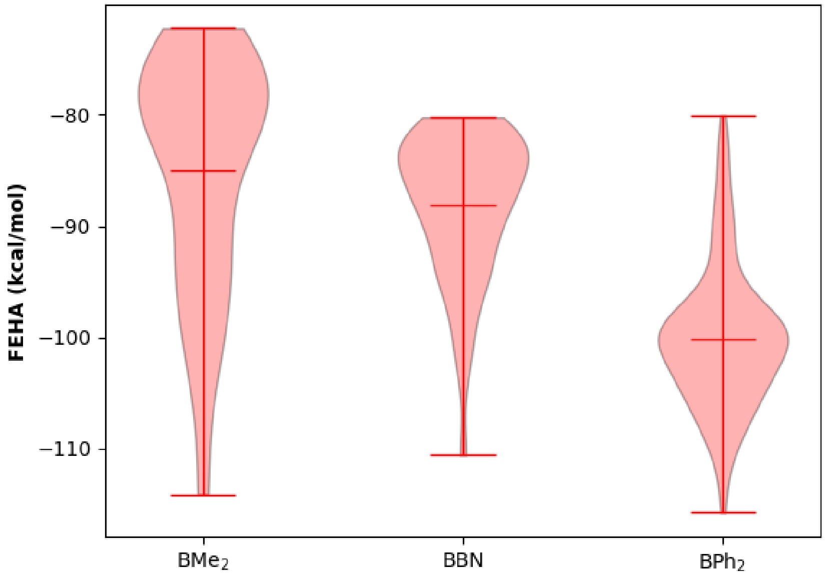

Figure S7. Distribution of the FEHA values for LPASs during screening based on chemical criteria.

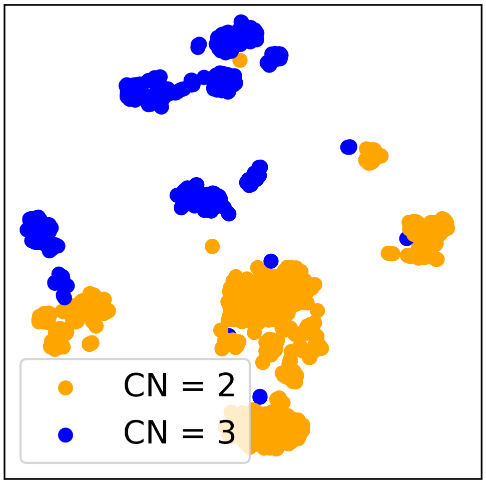

Figure S8. 2D t-SNE map of the chemical diversity of all LPASs featuring -BPh_2 as the LA fragment on the basis of atomic SLATM representation of the B and N centers. Each point corresponds to an LPAS colored by the coordination number (CN) of the nitrogen atom.

Table S2: FEPA and FEHA values of a few common experimentally used LAs and LBs. Mes = 2,4,6-$Me_3C_6H_2$, DBU = 1,8-Diazabicyclo[5.4.0]undec-7-ene, DABCO = 1,4-Diazabicyclo[2.2.2]octane.

LAs/LBs	FEPA (kcal/mol)	FEHA (kcal/mol)
**$B(C_6F_5)_3$**	–	-118.0
**$B(Mes)_3$**	–	-79.2
**DBU**	-252.6	–
**DABCO**	-230.6	–

S5: Machine learning model for porosity prediction

PLD data and framework are taken from Rosseinsky *et al.*,[36] switching the model from classification to regression. To this end, we use a Catboost model.[37] As in the original work by Rosseinsky *et al.*,[36] linkers are encoded as fingerprints using the Mordred package,[38] which are then fed to the gradient boosting model for training and prediction. The models were trained through 6000 iterations with a learning rate of 0.01 and the mean absolute error (MAE) as the loss function. The resulting model was subjected to 10-fold cross-validation and yielded an out-of-fold $R2 = 0.85$ and $MAE = 0.46$ Å, which we deemed sufficient for

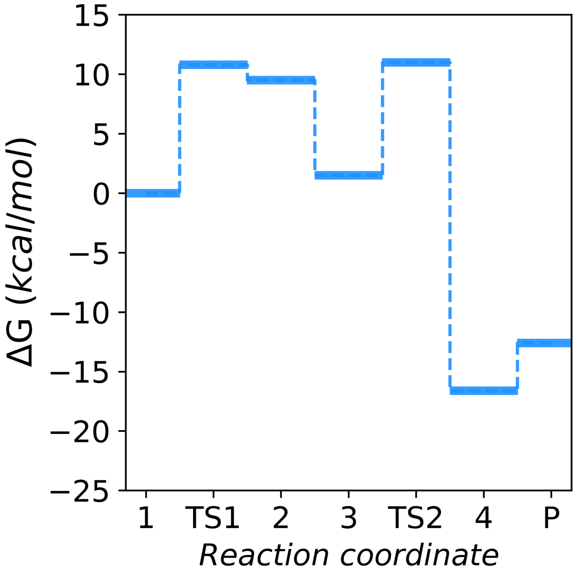

Figure S9. Free energy profile for the LPAS D.

our application.

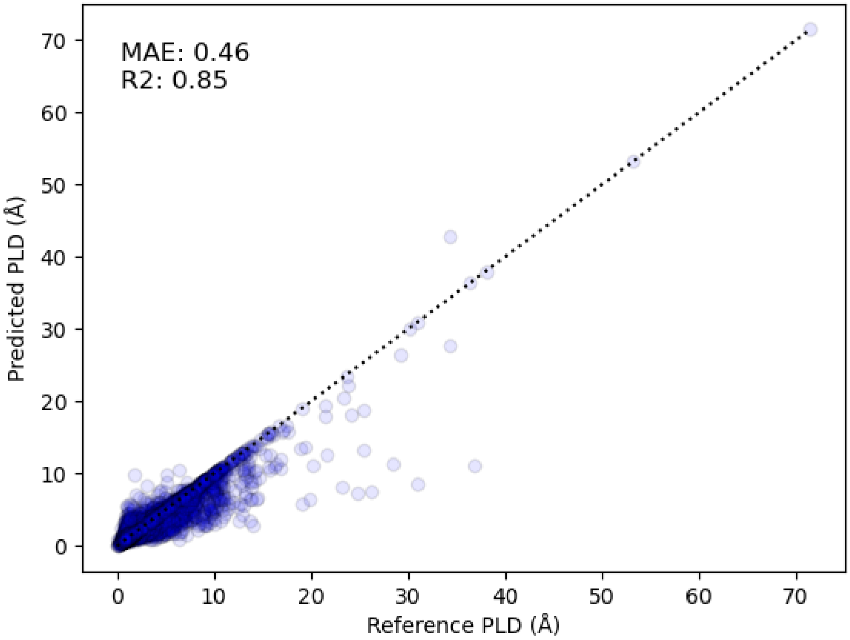

Figure S10. Parity plot obtained using the Catboost model to predict the PLD using the data from Rosseinsky *et al.*[36] Predicted points correspond to the out-of-the-fold points through 10-fold cross-validation.

S6: Structural analysis of MOFs with different topologies

We constructed a set of MOF structures by combining seven representative LPAS-containing linkers and six metal nodes. These linkers were chosen from among the top LPASs suggested by our pipeline. During the MOF construction, the choice of topologies was restricted to the top 100 options selected based on their occurrence in the DigiMOF database.[13] Since

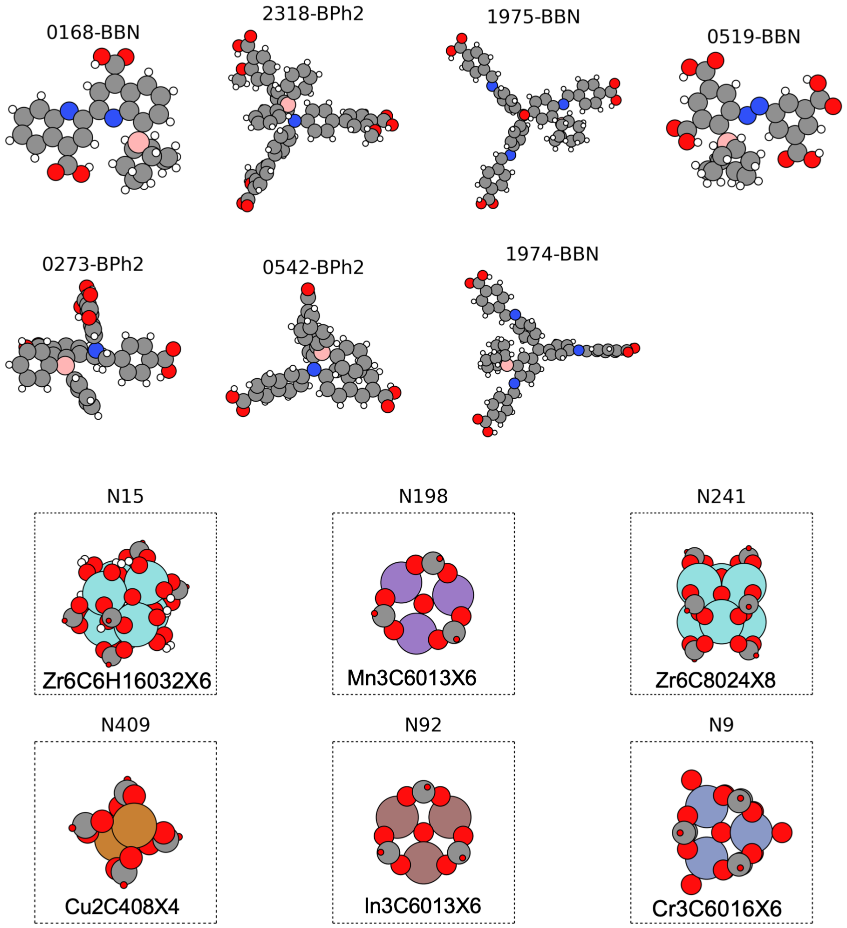

Figure S11. Selected LPAS-containing linkers (*top*) and metal nodes (*bottom*) used for MOF construction.

not all nodes, linkers, and topologies are compatible with each other due to coordination number or bonding distance mismatches between the metals and topologies, we initially constructed 97 MOFs following our structural criteria (see Computational details section S2). Subsequently, upon geometry optimization, we ended up with 43 MOFs featuring 13

different topologies. The histogram in Figure S12a shows the distribution of the PLD values in this set, which reveals that all MOFs satisfy the minimally required porosity criteria of PLD > 3.8 Å. More than 60% of the MOFs, particularly those with *rtl*, *apo*, *sit*, *stp*, and *the* topologies, present PLDs of over 12 Å. This implies that these LPASs can be incorporated within pores which are large enough to enable *in situ* functionalization, given the appropriate node and topology.[39] Figure S12b shows the averaged pore sizes for a few topologies, which are found at least three or more times in this set of MOFs. The variation of PLDs for a particular choice of linker is shown in Figure S12c which suggests that within this set, tritopic linkers lead to bigger PLDs compared to ditopic and tetratopic ones.

To examine the separation between the neighboring LPASs in the MOF structure, we calculated the geometric center for each pair of B and N atoms of an LPAS and evaluated the distance between all such centroids within the unit cell of the MOF. The minimum of those distances, denoted as minimum centroid distance (MCD), is used as a measure for the closeness between separate LPASs present within a given pore. Figure S12d shows a distribution of MCDs for the 43 MOFs. The results reveal that more than 93% (40 out of 43) of the MOFs possess MCD greater than 6 Å (roughly twice the average B-N distances in the highly active LPASs), indicating that the neighboring LPASs remain well-separated from each other and are not expected to inhibit each other's catalytic activity.

Therefore, we conclude that our pipeline - which includes a machine learning model to predict PLD from the metal and linker identity (see Section S5) - is able to (a) filter out inappropriate linkers (e.g. ones that are too small or have neighboring connection sites) that would lead to small pores and closely-spaced LPASs, and (b) propose linkers that are generally compatible with multiple topologies and metal nodes.

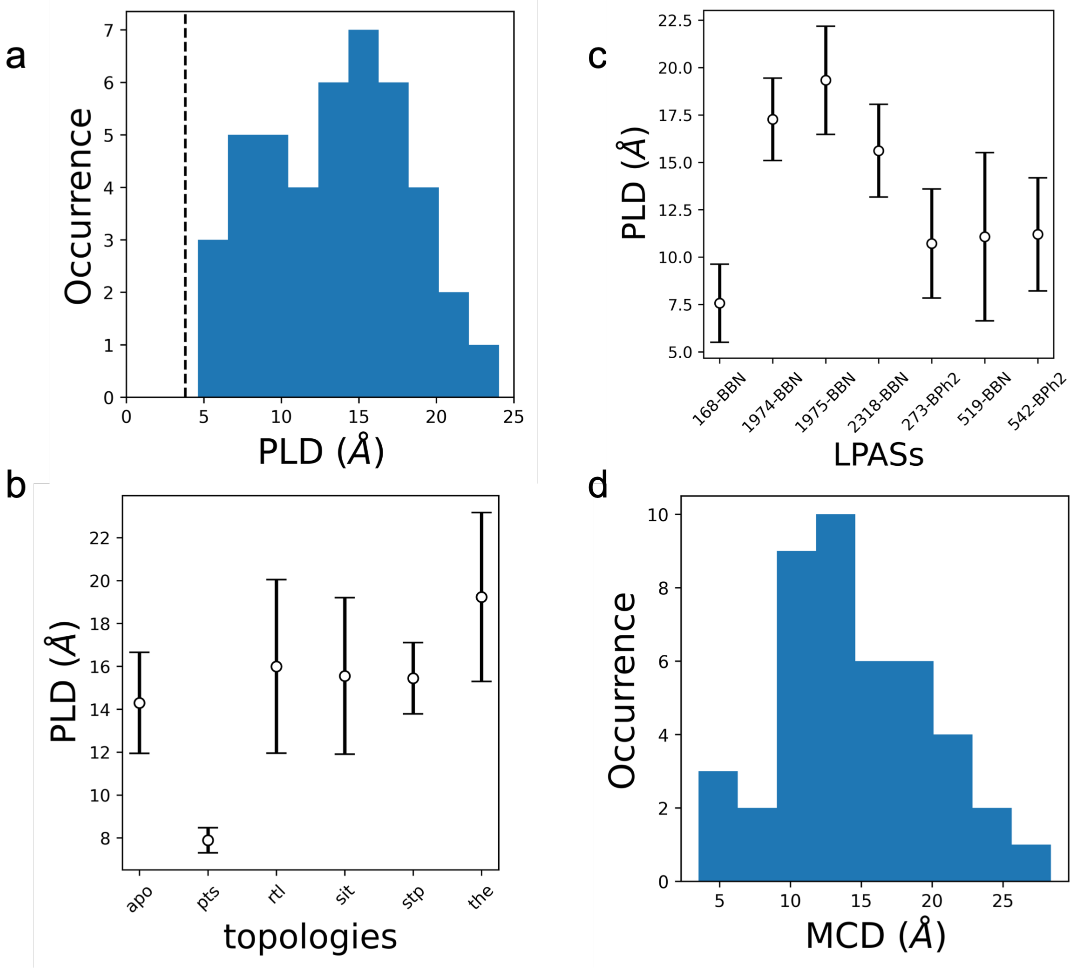

Figure S12. (a) Histogram of computed PLDs from 43 MOF structures. The black dashed line corresponds to the PLD threshold 3.8 Å. (b) The distribution of PLDs for MOFs constructed from topologies that appear at least three or more times in this set. The empty circle corresponds to the mean PLD while the whiskers correspond to the standard deviations. (c) The distribution of PLDs for MOFs constructed from seven LPAS-containing linkers. The empty circle corresponds to the mean PLD while the whiskers correspond to the standard deviations. (d) Histogram of computed MCDs.

S7: Comparison with the experimentally reported intramolecular FLPs for stoichiometric CO_2 hydrogenation

In 2015, Fontaine, Stephan, and co-workers reported a family of intramolecular borane-amine FLPs on ortho-substituted benzene scaffolds. Two starting compounds, 1-(NMe_2)-2-(B(2,4,5-$Me_3C_6H_2$)$_2$)-C_6H_4 and 1-(NMe_2)-2-(B(2,4,6-$Me_3C_6H_2$)$_2$)-C_6H_4 (**H** and

I, Figure S13), were shown to stoichiometrically reduce CO_2 to boron-bound formates, acetals and methoxides.[24] NMR and computational studies on **H** and **I** suggested that both undergo

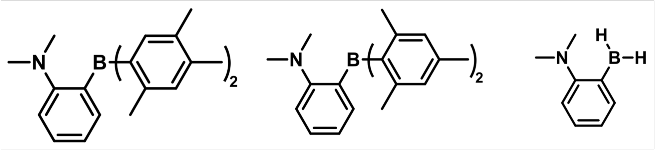

H I J

Figure S13. Structures of the experimentally reported FLPs **H-J**.

protodeborylation, in which both substituents on the boron center are lost following hydrogen activation. As a result, the likely active species of both **H** and **I** is in fact the -BH_2 derivative **J**. Based on the computed FEPA and FEHA values, J does not fall within the hotspot of the activity map in Figure 4c, indicating the lack of complementarity between acid and base centers (FEPA + FEHA = -302.1 kcal/mol). This explains the lack of catalytic turnover observed for this FLP. In contrast, it has been shown that appropriately matched Lewis pairs - chosen based on their computed FEPA and FEHA values - can lead to catalytic turnover for CO_2 reduction.[29] The predicted highly active LPASs with vicinally disposed B and N centers (**B**, **C**, **F**, **G**) , appropriately balance FEPA and FEHA. The computed descriptor values for FLP **J** along with closely related LPAS **B**, **C**, **F**, and **G** are reported in Table S3.

Table S3: Comparison of geometrical and chemical descriptors for the experimental FLP J and proposed LPASs B, C, F, and G.

**FLPs**	d **(Å),Φ(°)**	**FEHA, FEPA (kcal/mol)**
**J**	2.79, 114.0	-81.6, -220.5
**B**	2.70, 81.7	-81.3, -235.5
**C**	2.75, 68.4	-96.7, -220.8
**F**	2.84, 90.1	-100.6, -216.3
**G**	2.71, 88.3	-97.5,-219.2

References

(1) O'Boyle, N. M.; Banck, M.; James, C. A.; Morley, C.; Vandermeersch, T.; Hutchison, G. R. Open Babel: An open chemical toolbox. *J. Cheminf.* **2011**, *3*, 1–14.

(2) Bannwarth, C.; Ehlert, S.; Grimme, S. GFN2-xTB—An accurate and broadly parametrized self-consistent tight-binding quantum chemical method with multipole electrostatics and density-dependent dispersion contributions. *J. Chem. Theory. Comput.* **2019**, *15*, 1652–1671.

(3) Das, S.; Laplaza, R.; Blaskovits, J. T.; Corminboeuf, C. Mapping Active Site Geometry to Activity in Immobilized Frustrated Lewis Pair Catalysts. *Angew. Chem. Int. Ed.* **2022**, *61*, e202202727.

(4) Perdew, J. P.; Burke, K.; Ernzerhof, M. Generalized gradient approximation made simple. *Phys. Rev. Lett.* **1996**, *77*, 3865.

(5) Adamo, C.; Barone, V. Toward reliable density functional methods without adjustable parameters: The PBE0 model. *J. Chem. Phys.* **1999**, *110*, 6158–6170.

(6) Grimme, S.; Antony, J.; Ehrlich, S.; Krieg, H. A consistent and accurate ab initio parametrization of density functional dispersion correction (DFT-D) for the 94 elements H-Pu. *J. Chem. Phys.* **2010**, *132*, 154104.

(7) Grimme, S.; Ehrlich, S.; Goerigk, L. Effect of the damping function in dispersion corrected density functional theory. *J. Comput. Chem.* **2011**, *32*, 1456–1465.

(8) Weigend, F.; Ahlrichs, R. Balanced basis sets of split valence, triple zeta valence and quadruple zeta valence quality for H to Rn: Design and assessment of accuracy. *Phys. Chem. Chem. Phys.* **2005**, *7*, 3297–3305.

(9) Frisch, M. J.; Trucks, G. W.; Schlegel, H. B.; Scuseria, G. E.; Robb, M. A.;

Cheeseman, J. R.; Scalmani, G.; Barone, V.; Petersson, G. A.; Nakatsuji, H. et al. Gaussian 16 Revision A.03. 2016; Gaussian Inc. Wallingford CT.

(10) Funes-Ardoiz, I.; Paton, R. GoodVibes v2. 0.2. *DOI:http://doi.org/10.5281/zenodo.595246* **2019**, 291.

(11) Huang, B.; von Lilienfeld, O. A. Quantum machine learning using atom-in-molecule-based fragments selected on the fly. *Nat. Chem.* **2020**, *12*, 945–951.

(12) Lee, S.; Kim, B.; Cho, H.; Lee, H.; Lee, S. Y.; Cho, E. S.; Kim, J. Computational screening of trillions of metal–organic frameworks for high-performance methane storage. *ACS Applied Materials & Interfaces* **2021**, *13*, 23647–23654.

(13) Glasby, L. T.; Gubsch, K.; Bence, R.; Oktavian, R.; Isoko, K.; Moosavi, S. M.; Cordiner, J. L.; Cole, J. C.; Moghadam, P. Z. DigiMOF: a database of metal–organic framework synthesis information generated via text mining. *Chemistry of Materials* **2023**, *35*, 4510–4524.

(14) Rappé, A. K.; Casewit, C. J.; Colwell, K.; Goddard III, W. A.; Skiff, W. M. UFF, a full periodic table force field for molecular mechanics and molecular dynamics simulations. *Journal of the American chemical society* **1992**, *114*, 10024–10035.

(15) Gale, J. D.; Rohl, A. L. The general utility lattice program (GULP). *Molecular Simulation* **2003**, *29*, 291–341.

(16) Haranczyk, M.; Sethian, J. A. Automatic structure analysis in high-throughput characterization of porous materials. *Journal of chemical theory and computation* **2010**, *6*, 3472–3480.

(17) Te Velde, G. t.; Bickelhaupt, F. M.; Baerends, E. J.; Fonseca Guerra, C.; van Gisbergen, S. J.; Snijders, J. G.; Ziegler, T. Chemistry with ADF. *J. Comput. Chem.* **2001**, *22*, 931–967.

(18) Kühne, T. D.; Iannuzzi, M.; Del Ben, M.; Rybkin, V. V.; Seewald, P.; Stein, F.; Laino, T.; Khaliullin, R. Z.; Schütt, O.; Schiffmann, F. et al. CP2K: An electronic structure and molecular dynamics software package-Quickstep: Efficient and accurate electronic structure calculations. *J. Chem. Phys.* **2020**, *152*, 194103.

(19) Ernzerhof, M.; Scuseria, G. E. Assessment of the Perdew–Burke–Ernzerhof exchange-correlation functional. *J. Chem. Phys.* **1999**, *110*, 5029–5036.

(20) Krack, M. Pseudopotentials for H to Kr optimized for gradient-corrected exchange-correlation functionals. *Theor. Chem. Acc.* **2005**, *114*, 145–152.

(21) VandeVondele, J.; Hutter, J. Gaussian basis sets for accurate calculations on molecular systems in gas and condensed phases. *J. Chem. Phys.* **2007**, *127*, 114105.

(22) Laplaza, R. libarvo: library to compute molecular surfaces and volumes. https://github.com/rlaplaza/libarvo, (accessed 2023-02-01).

(23) Ashley, A. E.; Thompson, A. L.; O'Hare, D. Non-metal-mediated homogeneous hydrogenation of CO_2 to CH3OH. *Angew. Chem. Int. Ed.* **2009**, *48*, 9839–9843.

(24) Courtemanche, M.-A.; Pulis, A. P.; Rochette, É.; Légaré, M.-A.; Stephan, D. W.; Fontaine, F.-G. Intramolecular B/N frustrated Lewis pairs and the hydrogenation of carbon dioxide. *Chem. Commun.* **2015**, *51*, 9797–9800.

(25) Liu, L.; Vankova, N.; Heine, T. A kinetic study on the reduction of CO_2 by frustrated Lewis pairs: from understanding to rational design. *Phys. Chem. Chem. Phys.* **2016**, *18*, 3567–3574.

(26) Tran, S. D.; Tronic, T. A.; Kaminsky, W.; Heinekey, D. M.; Mayer, J. M. Metal-free carbon dioxide reduction and acidic C–H activations using a frustrated Lewis pair. *Inorg. Chim. Acta* **2011**, *369*, 126–132.

(27) Zhao, T.; Hu, X.; Wu, Y.; Zhang, Z. Hydrogenation of CO_2 to formate with H_2: transition metal-free catalyst based on a Lewis pair. *Angew. Chem. Int. Ed.* **2019**, *58*, 722–726.

(28) Wen, M.; Huang, F.; Lu, G.; Wang, Z.-X. Density functional theory mechanistic study of the reduction of CO_2 to CH_4 catalyzed by an ammonium hydridoborate ion pair: CO_2 activation via formation of a formic acid entity. *Inorg. Chem.* **2013**, *52*, 12098–12107.

(29) Das, S.; Turnell-Ritson, R. C.; Dyson, P. J.; Corminboeuf, C. Design of Frustrated Lewis Pair Catalysts for Direct Hydrogenation of CO_2. *Angew. Chem. Int. Ed.* **2022**, *61*, e202208987.

(30) Laplaza, R.; Das, S.; Wodrich, M. D.; Corminboeuf, C. Constructing and interpreting volcano plots and activity maps to navigate homogeneous catalyst landscapes. *Nat. Protoc.* **2022**, *17*, 2550–2569.

(31) Wodrich, M. D.; Busch, M.; Corminboeuf, C. Accessing and predicting the kinetic profiles of homogeneous catalysts from volcano plots. *Chem. Sci.* **2016**, *7*, 5723–5735.

(32) Wodrich, M. D.; Sawatlon, B.; Busch, M.; Corminboeuf, C. The Genesis of Molecular Volcano Plots. *Acc. Chem. Res.* **2021**, *54*, 1107–1117.

(33) Busch, M.; Wodrich, M. D.; Corminboeuf, C. A generalized picture of C–C cross-coupling. *ACS Catal.* **2017**, *7*, 5643–5653.

(34) Grimme, S.; Bannwarth, C.; Dohm, S.; Hansen, A.; Pisarek, J.; Pracht, P.; Seibert, J.; Neese, F. Fully automated quantum-chemistry-based computation of spin–spin-coupled nuclear magnetic resonance spectra. *Angew. Chem. Int. Ed.* **2017**, *56*, 14763–14769.

(35) Grimme, S.; Bannwarth, C.; Shushkov, P. A robust and accurate tight-binding quantum chemical method for structures, vibrational frequencies, and noncovalent interactions

of large molecular systems parametrized for all spd-block elements (Z= 1–86). *J. Chem. Theory Comput.* **2017**, *13*, 1989–2009.

(36) Pétuya, R.; Durdy, S.; Antypov, D.; Gaultois, M. W.; Berry, N. G.; Darling, G. R.; Katsoulidis, A. P.; Dyer, M. S.; Rosseinsky, M. J. Machine-Learning Prediction of Metal–Organic Framework Guest Accessibility from Linker and Metal Chemistry. *Angew. Chem. Int. Ed.* **2022**, *61*, e202114573.

(37) Prokhorenkova, L.; Gusev, G.; Vorobev, A.; Dorogush, A. V.; Gulin, A. CatBoost: unbiased boosting with categorical features. 2019.

(38) Moriwaki, H.; Tian, Y.-S.; Kawashita, N.; Takagi, T. Mordred: a molecular descriptor calculator. *Journal of cheminformatics* **2018**, *10*, 1–14.

(39) Zhang, X.; Huang, Z.; Ferrandon, M.; Yang, D.; Robison, L.; Li, P.; Wang, T. C.; Delferro, M.; Farha, O. K. Catalytic chemoselective functionalization of methane in a metal- organic framework. *Nature Catalysis* **2018**, *1*, 356–362.
